# The Interplay between Atherosclerosis and Cancer: Breast Cancer Cells Increase the Expression of Endothelial Cell Adhesion Markers

**DOI:** 10.3390/biology12070896

**Published:** 2023-06-22

**Authors:** Alessandro Scalia, Lesly Doumani, Nadège Kindt, Fabrice Journé, Anne Trelcat, Stéphane Carlier

**Affiliations:** 1 Department of Cardiology, Research Institute for Health Sciences and Technology, University of Mons (UMONS), 7000 Mons, Belgium; alessandro.scalia@umons.ac.be (A.S.); lesly.doumanimetsim@student.umons.ac.be (L.D.); nadege.kindt@hubruxelles.be (N.K.); anne.trelcat@umons.ac.be (A.T.); 2 Department of Clinical and Experimental Oncology, Institut Jules Bordet, Université Libre de Bruxelles, 1000 Brussels, Belgium; fabrice.journe@umons.ac.be

**Keywords:** breast cancer, conditioned medium, atherosclerosis, oxLDL, THP-1, oxidation

## Abstract

**Simple Summary:**

Cardiovascular diseases and cancer are the two most common causes of death worldwide. Although evidence shows a relationship between these two pathologies, there remain many unknown factors regarding their connection. We investigated the impact of the cancer environment on the initiation of atherosclerosis, which is a major contributor to cardiovascular diseases. We observed that the cancer cell medium influences the expression of markers involved in the adhesion of monocytes and endothelial cells, leading to the initiation of atherosclerosis. Additionally, we found that cancer cells have the capacity to oxidize low-density lipoproteins, thereby increasing the likelihood of cholesterol plaque formation in the arteries. In conclusion, our results suggest that cancer could promote atherosclerosis through the increased expression of endothelial cell markers and oxidation of low-density lipoproteins. These findings should be confirmed through clinical studies.

**Abstract:**

Cardiovascular diseases are the leading causes of death worldwide, closely followed by cancer. To investigate the impact of breast cancer cell lines (SKBR3, MCF-7, and MDA-MB-231) on endothelial cell adhesion, a blended medium containing 30% breast-cancer-conditioned medium was prepared. This medium was then exposed to human umbilical vein endothelial cells (HUVECs) and monocytes (THP-1) for 48 h. Homemade oxidized low-density lipoproteins (oxLDL) were optionally added to the blended medium. Immunofluorescence was performed to assess the expression of E-selectin, connexin-43, and ICAM-1 on HUVECs, as well as LOX-1, CD36, and CD162 on THP-1. Additionally, unoxidized LDL was exposed to the three breast cancer cell lines for 48 h, and the formation of oxLDL was quantified. Our results revealed an upregulation of all six adhesion markers involved in the initiation of atherosclerosis when HUVECs and THP-1 were exposed to the breast-cancer-conditioned medium. Furthermore, this expression was further increased by exposure to oxLDL. We also observed a significant elevation in oxLDL levels when LDL was exposed to breast cancer cells. In conclusion, our findings successfully demonstrate an increased LDL oxidation in the presence of breast cancer cells, accompanied by an augmented expression of receptors involved in atherosclerosis initiation. These findings shed new light on the clinically observed interplay between atherosclerosis and cancer.

## 1. Introduction

Cardiovascular diseases (CVDs) are the leading causes of death worldwide, causing 32% of all deaths [1], closely followed by cancer, which causes nearly 16% of deaths each year [2]. In the European Union, breast cancer is the most common cancer in females, representing 29.2% of all cancers in females [3]. In 2018, breast cancer was the leading cause of death from cancer in women [4]. As mortality rates decrease with the improvement of screening and treatment, breast cancer is reported to be in second place after lung cancer [5]. In recent years, there is increasing substantial evidence that CVDs and cancer are linked together, sharing many comorbidities and risk factors such as age, sedentary lifestyle, smoking habit, a fat- and carbohydrate-rich diet, hypertension, or diabetes mellitus [6]. Furthermore, cancer survivors have a significantly higher risk of CVDs compared to adults without cancer, with an incidence rate of 2300 per 100,000 person-years versus 1200, respectively [7].

Oxidative stress and neoangiogenesis seem to be the cornerstones linking them. Atherosclerosis represents the main actor leading CVDs under the spotlight. Its initiation starts with the alteration of the endothelium. The increased concentration and duration of low-density lipoproteins (LDL) in the subendothelium leads to an increased oxidation rate [8,9]. The resulting oxidized low-density lipoproteins (oxLDL) promote the production of interleukin-1β (IL-1β) mainly in endothelial cells and macrophages [10]. Indeed, after binding to lectin-type oxidized LDL receptor 1 (LOX-1), oxLDL increases reactive oxygen species (ROS) and decreases nitric oxide (NO) release, resulting in the activation of epithelial-to-mesenchymal transition transcription factors (EMT-TFs), inflammatory signaling (IL-6, IL-8, and IL-1β), and also the hypoxia pathways as vascular endothelial growth factor (VEFG) and hypoxia-inducible factor-1α (HIF-1α) [11].

**Figure 1:**
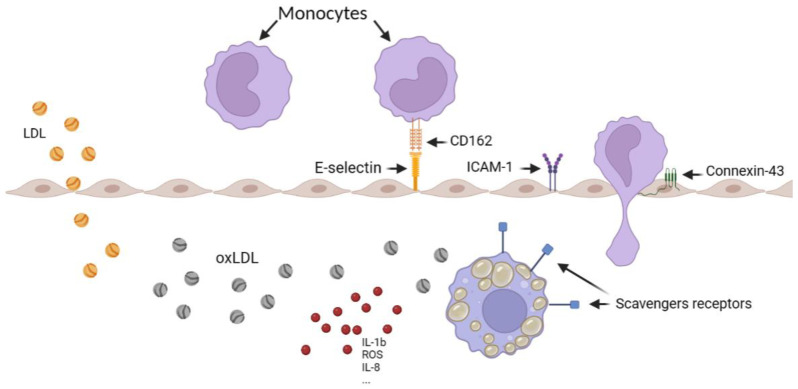
Schematic representation of monocyte recruitment in the artery, illustrating the various steps involved, including LDL oxidation, foam cell formation, and the overexpression of scavenger receptors. The figure was created using Biorender.com.

Monocyte recruitment through the endothelium is a crucial step in the initiation of atherosclerosis and can be separated into several main steps including the capture, the rolling, the adhesion, the crawling, and the extravasation, all initiated by different actors (Figure 1). P-selectin glycoprotein-ligand 1 (PSGL-1 or CD162) on the surface of monocytes binding E-selectin and P-selectin on the surface of endothelial cells trigger the capture and the rolling step, helped by the endothelial vascular cell-adhesion molecule-1 (VCAM-1) and intercellular adhesion molecule-1 (ICAM-1) for the adhesion. The transmigration is mediated by connexins (as connexin-43 or connexin-37) and by junctional adhesion molecules (predominantly JAM-A and JAM-C) [12,13,14]. In atherosclerosis, all these factors appear to be overexpressed, explaining the large number of monocytes in the plaque [12].

Moreover, scavenger receptors such as LOX-1, as well as cluster of differentiation 36 (CD36), the scavenger receptor type A (SR-A), and the scavenger receptor class B type 1 (SRB-1) on the macrophage surface into the media induce oxLDL capture, transforming them into foam cells. Such cells release even more cytokines and growth factors [15], decreasing their migration abilities to retaining them into the plaque in formation [16].

Interestingly, besides sharing the same risk factors, atherosclerosis and cancer share the same biochemical alterations during their initiations, which could explain the ineluctable bond between them [17].

The influence of lipoproteins on cancer cells has been investigated for a while. In 1989, Rotheneder et al. studied the effect of LDL and high-density lipoproteins (HDL) on the proliferation of breast cancer cells in vitro, showing that LDL stimulated the proliferation of hormone-independent lines but had no effect on hormone-sensitive lines [18]. High LDL plasma levels were found to be correlated with human epidermal growth factor receptor-2 (HER2)-Neu positive breast cancer [19]. More recently, oxLDL showed a dose-dependent stimulation of proliferation mediated by the proinflammatory PI3K/Akt pathway [20]. Finally, OxLDL lecithin-like receptor 1 (OLR1), the main receptor for oxLDL internalization, is found to be overexpressed in human breast cancer cells and positively correlated to tumor stage and grade [21].

From a more clinical point of view, Van’t Klooster et al. recently reported a correlation between cardiovascular diseases and the incidence of new cancer, with inflammation being the common underlying factor. In their study, a 1 mg/L increase in C-reactive protein (CRP) among 7178 patients with stable cardiovascular diseases was associated with an increased incidence of new cancer (HR 1.07, 95%CI 1.04–1.09), particularly for lung cancer (HR 1.16, 95%CI 1.10–1.22) [22]. Furthermore, cardiovascular diseases exhibited a recurrence rate of 1.53% per person per year when CRP levels were below 2 mg/L, which then increased to 3.3% per person per year when CRP levels ranged from 8–10 mg/L [22]. Raza et al. also observed elevated plasma LDL levels in patients with advanced-stage breast cancer compared to those with grade I cancer or healthy women [23]. Lastly, Pope et al. recently demonstrated increased subclinical atherosclerosis in breast cancer survivors using PET-CT imaging [24]. Despite these studies, controversies still exist. Indeed, previous research has indicated that lipid-lowering therapies were associated with a reduced risk of breast cancer [25], while other studies have not been able to confirm this protective effect against breast cancer risk [26].

To increase the understanding of the relationships between breast cancer and atherosclerosis, we sought to investigate the influence of the breast cancer environment on the expression of endothelial cell adhesion, which represents the first step of atherosclerosis, and investigate the potential oxidative effect of breast cancer cells on LDL.

## 2. Materials and Methods

### 2.1. oxLDL Production

In order to investigate the influence of oxLDL on human umbilical vein endothelial cells (HUVECs) and on human leukaemia monocytic cell line (THP-1), we used our “homemade” oxLDL, where we isolated and oxidated LDL using our previously described protocol [27]. LDL and VLDL were first isolated from human plasma by serial precipitation and centrifugation steps. LDL were separated from VLDL by high-performance liquid chromatography in an aqueous size-exclusion chromatography column. Then, LDL was oxidated by 5 µM CuSO_4_ incubation while agarose gel electrophoresis was assessed to control the quality of the oxidation [27].

### 2.2. Cell Culture

Three breast cancer cell lines, human umbilical vein endothelial cell (HUVEC), and human leukaemia monocytic cell (THP-1) were used in these experiments (Table 1) [28].

The breast cancer cell lines were grown in Dulbecco’s Modified Eagle Medium (DMEM, Lonza, Verviers, Belgium) supplemented with 10% fetal bovine serum (FBS), 2% L-Glutamine, 1% non-essential amino acids, and 1% Penicillin/Streptomycin (all provided by Gibco™ Life Technologies, Paisley, UK).

Human umbilical vein endothelial cell (HUVEC) line was grown on Endothelial Cell Growth Base Media (R&D Systems, Bio-Techne, Minneapolis, MN, USA) supplemented with 1% Penicillin/Streptomycin (Gibco™ Life Technologies, Paisley, UK) and provided Endothelial Cell Growth Supplement (50×).

Human leukaemia monocytic cell (THP-1) line was grown in Roswell Park Memorial Institute 1640 medium (RPMI, Gibco™ Life Technologies, Paisley, UK) supplemented with 10% FBS, 5% L-Glutamine, and 1% Penicillin/Streptomycin (all provided by Gibco™ Life Technologies, Paisley, UK).

Routine cell cultures were performed at 37 °C in a humidified cell incubator under 5% CO_2_.

### 2.3. Enzyme-Linked Immunosorbent Assay (ELISA)

When 70% of T25 flask confluence was reached, SK-BR3, MDA-MB-231, and MCF-7 medium were replaced with fresh medium containing 10% of delipidated and decomplemented Charcoal-Stripped FBS (Labconsult SA-NV, Brussels, Belgium), and unoxidized LDL was added to the medium at 50 µg/mL. After 48 h, the medium containing added LDL was collected and stored, protected from the light at 4 °C until the next steps (corresponding to the not-absorbed LDL).

Then, the cell monolayer was rinsed two times with Dulbecco’s phosphate-Buffer (DPBS, Lonza, Verviers, Belgium). Next, 250 µL of lysis reagent (M-PER™ Mammalian Protein Extraction Reagent, ThermoFisher Scientific, Waltham, MA, USA) and 10 µL/mL of protease and phosphatase inhibitors (Halt™ Protease and Phosphatase Inhibitor Cocktail (100×), ThermoFisher Scientific, Waltham, MA, USA) were added in each T25 flask during 5 min incubation on ice. Cells were scraped, collected, vortexed, and incubated for 15 min on ice.

Finally, a last step of centrifugation at 13,000 rpm for 10 min at 4 °C allows us to recover the supernatant containing the intracellular content.

oxLDL quantitative measurements were assessed using a commercial ELISA kit (Cusabio, Houston, TX, USA) following manufacturer instructions. Reaction absorbance was analyzed with VersaMax Microplate Reader (Molecular Devices, San José, CA, USA) at 540 nm.

### 2.4. Immunofluorescence Microscopy on Human Umbilical Vein Endothelial Cells (HUVECs)

HUVECs were plated on sterile round glass coverslips coated beforehand with gelatin in a 12-well dish at 120,000 cells per well for 24 h. At the same time, with 70% confluence on T25 flasks, SK-BR3, MDA-MB-231, and MCF-7 medium were replaced with serum-free medium for 24 h.

The next day, the breast-cancer-conditioned medium was collected, centrifugated at 1500 rpm for 5 min to eliminate cells debris, then filtrated with Acrodisc^®^ Syringe Filter 0.2 µm with Supor^®^ Membrane (Pall Life Sciences, New York, NY, USA). HUVEC medium was replaced by a blend of 30% breast-cancer-conditioned medium and 70% fresh HUVEC medium. Negative control conditions contained a blend of 30% fresh serum-free DMEM and 70% fresh HUVEC medium. The two last conditions contained the blended conditions described, previously added with 10 µg/mL of “homemade” oxLDL. Each condition was performed in duplicate. After 48 h, cells were fixed with 4% paraformaldehyde in Phosphate-buffered saline 1× (PBS, Gibco Life Technologies, Paisley, UK) for 10 min at 4 °C and 5 min at room temperature (RT).

For anti-Connexin-43 primary antibody (Cell Signaling Technology, Danvers, MA, USA), the fixed cells were blocked for one hour at RT in 5% Normal Goat Serum (NGS) and 0.3% Triton-X100 in PBS. Then, anti-Connexin-43 antibody was diluted at 1/200 in a dilution buffer containing 1% Bovine Serum Albumin (BSA) and 0.3% Triton-X100 in PBS and applied on cells overnight at 4 °C. After several rinses with PBS, an anti-rabbit IgG antibody coupled with Alexa 488 was exposed on cells for 30 min (ThermoFisher Scientific, Waltham, MA, USA), diluted in the same solution as the primary antibody.

For anti-InterCellular Adhesion Molecule 1 (ICAM-1) primary antibody (Merk Sigma, Darmstadt, Germany), the fixed cells were permeabilized for 10 min at RT in a buffer containing 0.1% Trition-X100 in PBS, then for one hour at RT in a blocking buffer containing 5% NGS in PBS. Then, anti-ICAM-1 primary antibody was diluted at 1/100 in the blocking buffer and applied on cells overnight at 4 °C. After several rinses with PBS, an anti-rabbit IgG antibody coupled with Alexa 488 was exposed on cells for 30 min (ThermoFisher Scientific, Waltham, MA, USA), diluted in the same solution as the primary antibody.

For anti-E-selectin primary antibody (Merk Sigma, Darmstadt, Germany), the fixed cells were blocked for one hour at RT in 2% BSA and 0.3% Trition-X100 buffer. Then, anti-E-selectin primary antibody was diluted at 1/500 in the blocking buffer and applied on cells overnight at 4 °C. After several rinses with PBS, an anti-mouse IgG antibody coupled with Alexa 455 was exposed on cells for 30 min (ThermoFisher Scientific, Waltham, MA, USA), diluted in the same solution as the primary antibody.

After final rinses with PBS and deionized water, the coverslips were mounted onto glass slides using a commercial anti-fading medium (ProLong™Gold antifade reagent with DAPI, Invitrogen, ThermoFisher Scientific, Waltham, MA, USA). Confocal microscopy observations were carried out using a Nikon Eclipse Ti2-E inverted microscope (Nikon, Tokyo, Japan). Mean Fluorescence Intensity (MFI) was observed using ImageJ software.

### 2.5. Immunofluorescence Microscopy on Human Leukaemia Monocytic Cell Line (THP-1)

THP-1 were plated on sterile round glass coverslips in a 12-well dish at 200,000 cells per well for 24 h in RPMI medium containing Phorbol-12-myristate-13-acetate (PMA) at 100 ng/mL in order to induce macrophage polarization in state M0 [29]. At the same time, with 70% confluence on T25 flasks, SK-BR3, MDA-MB-231, and MCF-7 medium were replaced with serum-free medium for 24 h.

The next day, the breast-cancer-conditioned medium was collected, centrifugated, and filtrated as described previously. THP-1 medium was replaced by a blend of 30% breast-cancer-conditioned medium and 70% fresh THP-1 medium containing 10% of Charcoal-Stripped FBS (Labconsult SA-NV, Brussels, Belgium). Negative control conditions contained a blend of 30% fresh serum-free DMEM and 70% fresh RPMI medium containing 10% of Charcoal-Stripped FBS. The two last conditions contained the blended conditions described, previously added with 15 µg/mL of “homemade” oxLDL. Each condition was repeated in duplicate. After 48 h, cells were fixed with 4% paraformaldehyde in PBS for 10 min at 4 °C and 5 min at RT.

For anti-LOX-1 primary antibody (ThermoFisher Scientific, Waltham, MA, USA), the fixed cells were blocked for one hour at RT in 5% NGS and 0.3% Triton-X100 in PBS. Then, anti-LOX-1 antibody was diluted at 1/1000 in blocking solution and applied on cells overnight at 4 °C. After several rinses with PBS, an anti-rabbit IgG antibody coupled with Alexa 488 was exposed on cells for 30 min (ThermoFisher Scientific, Waltham, MA, USA), diluted in blocking solution.

For anti-CD36 primary antibody (Merk Sigma, Darmstadt, Germany), the fixed cells were blocked for one hour at RT in 2% BSA and 0.3% Triton-X100 in PBS. Then, anti-CD36 antibody was diluted at 1/100 in blocking solution and applied on cells overnight at 4 °C. After several rinses with PBS, an anti-rabbit IgG antibody coupled with Alexa 488 was exposed on cells for 30 min (ThermoFisher Scientific, Waltham, MA, USA), diluted in blocking solution.

For anti-CD162 primary antibody (Bio-Techne Corporation, Minneapolis, MN, USA), the fixed cells were blocked for one hour at RT in 5% NGS and 0.3% Triton-X100 in PBS. Then, anti-CD162 antibody was diluted at 1/20 in blocking solution and applied on cells overnight at 4 °C. After several rinses with PBS, an anti-mouse IgG antibody coupled with Alexa 455 was exposed on cells for 30 min (ThermoFisher Scientific, Waltham, MA, USA), diluted in blocking solution.

After final rinses with PBS and deionized water, the coverslips were mounted onto glass slides using a commercial anti-fading medium (ProLong™Gold antifade reagent with DAPI, Invitrogen, ThermoFisher Scientific, Waltham, MA, USA). Confocal microscopy observations were carried out using a Nikon Eclipse Ti2-E inverted microscope (Nikon, Tokyo, Japan). Mean Fluorescence Intensity (MFI) was observed using ImageJ software.

### 2.6. Statistical Analysis

Statistical analysis was performed with SPSS^®^ Statistics version 23 software (IBM^®^, Armonk, NY, USA). Student’s *t*-test was used, after confirming the applicability of parametric analyses using the Shapiro–Wilk test. Data were expressed as means ± SD with a *p* ≤ 0.05 value to indicate a statistically significant difference.

## 3. Results

### 3.1. LDL Oxidation under Breast Cancer Cell Exposition

**Figure 2:**
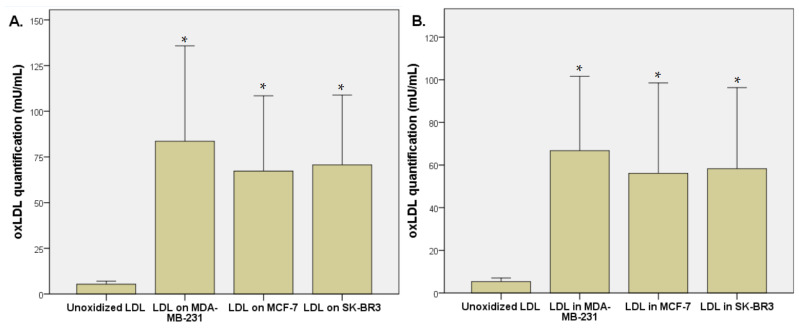
(**A**) oxLDL quantification (µU/mL) by ELISA method after 48 h exposition of unoxidized LDL on breast cancer cells (MDA-MB-231, MCF-7, and SK-BR3). (**B**) Intracellular oxLDL quantification (µU/mL) by ELISA method after 48 h exposition of unoxidized LDL on breast cancer cells (MDA-MB-231, MCF-7, and SK-BR3) (*n* = 6). * Represents the data reaching statistical significance (*p* < 0.05).

We observed an increased oxidation level of unoxidized LDL after its exposition for 48 h with breast cancer cells (MDA-MB-231, MCF-7, and SK-BR3). The unoxidized LDL level was 5.3 ± 1.6 mU/mL of oxLDL, that represents the level of spontaneous oxidation. oxLDL levels increased to 83.6 ± 49.8 mU/mL after MDA-MB-231 exposition (*p* = 0.013), 67.3 ± 39.3 mU/mL after MCF-7 exposition (*p* = 0.014), and 70.7 ± 36.4 mU/mL after SK-BR3 exposition (*p* = 0.006), demonstrating the ability of breast cancer cells to oxidize LDL (Figure 2A). Intracellular oxLDL levels reached 66.8 ± 33.2 µU/mL after MDA-MB-231 exposition (*p* = 0.007), 56.1 ± 40.4 mU/mL after MCF-7 exposition (*p* = 0.031), and 58.3 ± 36.3 mU/mL after SK-BR3 exposition (*p* = 0.014) (Figure 2B).

### 3.2. Endothelial Adhesion Markers in HUVEC upon Breast Cancer Environment Exposure

**Figure 3:**
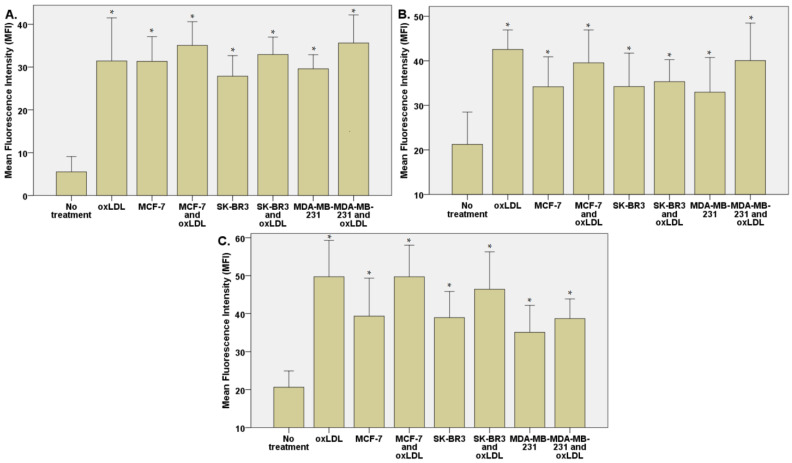
(**A**) E-selectin, (**B**) ICAM-1, and (**C**) Connexin-43 expressions in HUVECs were evaluated after a 48 h treatment with 30% breast cancer cell medium (following 24 h of incubation on breast cancer cells) and homemade oxLDL. * represents statistical significance.

**Figure 4:**
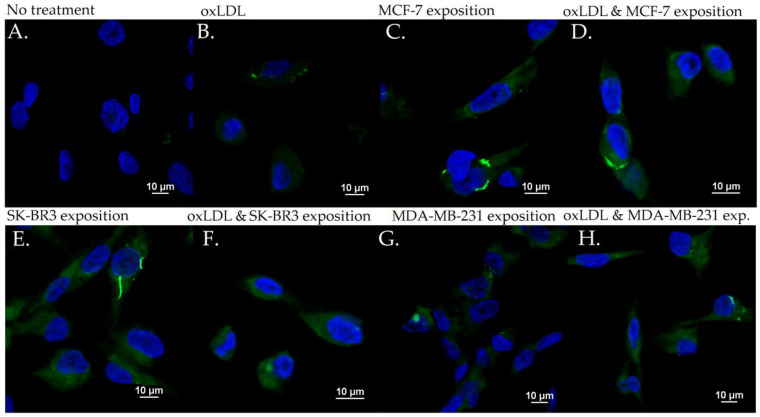
E-selectin expression in HUVEC with (**A**) no treatment, (**B**) oxLDL exposure for 48 h, (**C**) 30% MCF-7 medium exposure for 48 h, (**D**) oxLDL plus 30% MCF-7 medium exposure for 48 h, (**E**) 30% SK-BR3 medium exposure for 48 h, (**F**) oxLDL plus 30% SK-BR3 medium exposure for 48 h, (**G**) 30% MDA-MB-231 medium exposure for 48 h, and (**H**) oxLDL plus 30% MDA-MB-231 medium exposure for 48 h (*n* = 10).

The exposition of the breast cancer cell medium on HUVEC for 48 h results in a significant increase of endothelial adhesion markers. Indeed, we observed an E-selectin mean fluorescence intensity (MFI) of 5.53 for non-treated cells (NT). The oxLDL condition shows an MFI of 31.41 (*t*-test, *p* = 0.001), MCF-7 exposure shows an MFI of 31.34 (*t*-test, *p* < 0.001), SK-BR3 exposure shows an MFI of 27.87 (*t*-test, *p* < 0.001), and MDA-MB-231 exposure shows an MFI of 29.60 (*t*-test, *p* < 0.001). When adding oxLDL to an MCF-7 medium exposure, MFI was 35.07 (*t*-test, *p* < 0.001), while oxLDL in the SK-BR3 condition shows an MFI of 32.94 (*t*-test, *p* < 0.001) and oxLDL in the MDA-MB-231 condition shows an MFI of 35.62 (*t*-test, *p* < 0.001). There are no significant differences between the MCF-7 and MCF-7 plus oxLDL conditions (*t*-test, *p* = 0.08), nor between the MDA-MB-231 and MDA-MB-231 plus oxLDL conditions (*t*-test, *p* = 0.096). However, adding oxLDL to SK-BR3 reached statistical significance (*t*-test, *p* = 0.019) (Figure 3A and Figure 4).

**Figure 5:**
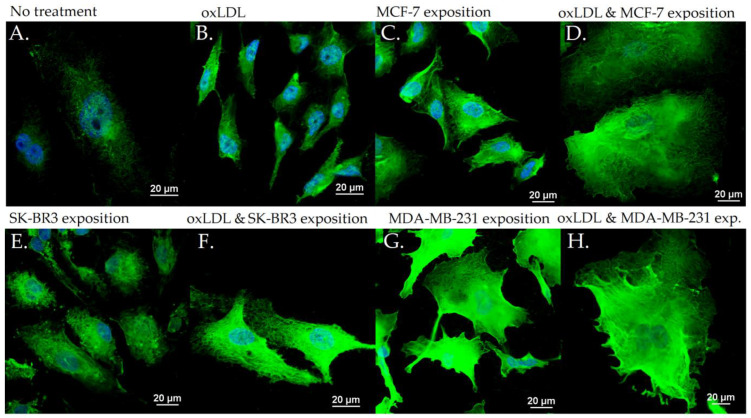
ICAM-1 expression in HUVEC with (**A**) no treatment, (**B**) oxLDL exposure for 48 h, (**C**) 30% MCF-7 medium exposure for 48 h, (**D**) oxLDL plus 30% MCF-7 medium exposure for 48 h, (**E**) 30% SK-BR3 medium exposure for 48 h, (**F**) oxLDL plus 30% SK-BR3 medium exposure for 48 h, (**G**) 30% MDA-MB-231 medium exposure for 48 h, and (**H**) oxLDL plus 30% MDA-MB-231 medium exposure for 48 h (*n* = 10).

Regarding the ICAM-1 expression, NT cells show an MFI of 21.24 whereas oxLDL exposition shows an MFI of 42.55 (*t*-test, *p* = 0.001). MCF-7 exposure shows an MFI of 34.18 (*t*-test, *p* = 0.009), SK-BR3 exposure shows an MFI of 34.23 (*t*-test, *p* = 0.038) and MDA-MB-231 shows an MFI of 32.96 (*t*-test, *p* = 0.036). When adding oxLDL to the MCF-7 medium exposure, we observed an MFI of 39.56 (*t*-test, *p* < 0.001). oxLDL added to the SK-BR3 medium shows an MFI of 34.23 (*t*-test, *p* = 0.012) whereas oxLDL to the MDA-MB-231 medium shows an MFI of 40.06 (*t*-test, *p* = 0.003). No significant differences were highlighted between MCF-7, SK-BR3, and MDA-MB-231 and the condition with the addition of oxLDL (*t*-test, respectively, *p* = 0.341, *p* = 0.796, and *p* = 0.162) (Figure 3B and Figure 5).

**Figure 6:**
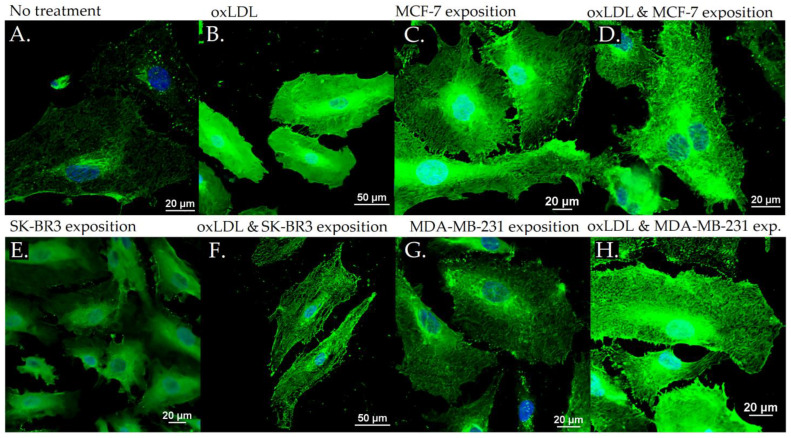
Connexin-43 expression in HUVEC with (**A**) no treatment, (**B**) oxLDL exposure for 48 h, (**C**) 30% MCF-7 medium exposure for 48 h, (**D**) oxLDL plus 30% MCF-7 medium exposure for 48 h, (**E**) 30% SK-BR3 medium exposure for 48 h, (**F**) oxLDL plus 30% SK-BR3 medium exposure for 48 h, (**G**) 30% MDA-MB-231 medium exposure for 48 h, and (**H**) oxLDL plus 30% MDA-MB-231 medium exposure for 48 h (*n* = 10).

MCF-7 exposure showed an MFI of 39.33 (*t*-test, *p* = 0.001), SK-BR3 exposure showed an MFI of 38.94 (*t*-test, *p* < 0.001), and MDA-MB-231 exhibited an MFI of 35.07 (*t*-test, *p* = 0.003). Upon the addition of oxLDL to the MCF-7 medium, we observed an MFI of 49.70 (*t*-test, *p* < 0.001). Similarly, oxLDL added to the SK-BR3 medium resulted in an MFI of 46.43 (*t*-test, *p* < 0.001), while oxLDL added to the MDA-MB-231 medium showed an MFI of 38.68 (*t*-test, *p* < 0.001). There were no significant differences between the MCF-7 and MCF-7 plus oxLDL conditions (*t*-test, *p* = 0.095), nor between the MDA-MB-231 and MDA-MB-231 plus oxLDL conditions (*t*-test, *p* = 0.4). However, adding oxLDL to the SK-BR3 medium reached statistical significance (*t*-test, *p* = 0.01) (Figure 3C and Figure 6).

Regarding the Connexin-43 expression, NT cells exhibited a mean fluorescence intensity (MFI) of 20.63, whereas exposure to oxLDL resulted in an MFI of 49.72 (*t*-test, *p* < 0.001).

### 3.3. Scavenger Receptors and Adhesive Marker in THP-1 Cells

**Figure 7:**
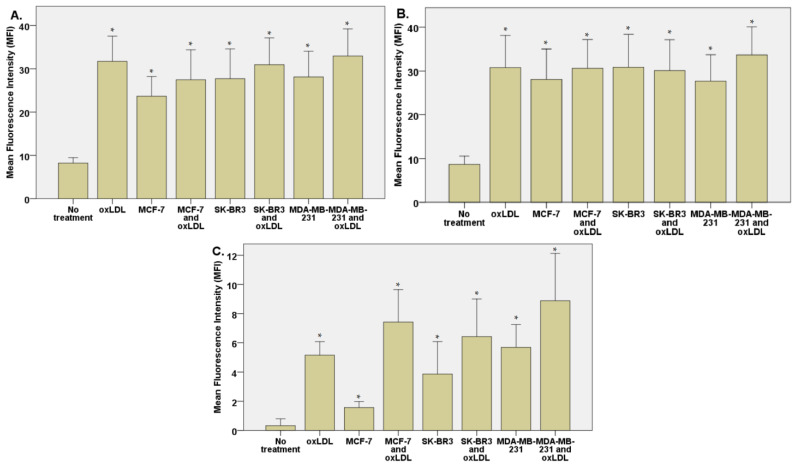
(**A**) LOX-1 expression in THP-1 cells treated, or not, for 48 h with 30% breast cancer cell medium (24 h incubation on breast cancer cells) and homemade oxLDL. (**B**) CD36 expression in THP-1 cells treated, or not, for 48 h with 30% breast cancer cell medium (24 h incubation on breast cancer cells) and homemade oxLDL. (**C**) CD162 expression in THP-1 treated, or not, for 48 h with 30% breast cancer cell medium (24 h incubation on breast cancer cells) and homemade oxLDL (*n* = 10). * represents statistical significance.

**Figure 8:**
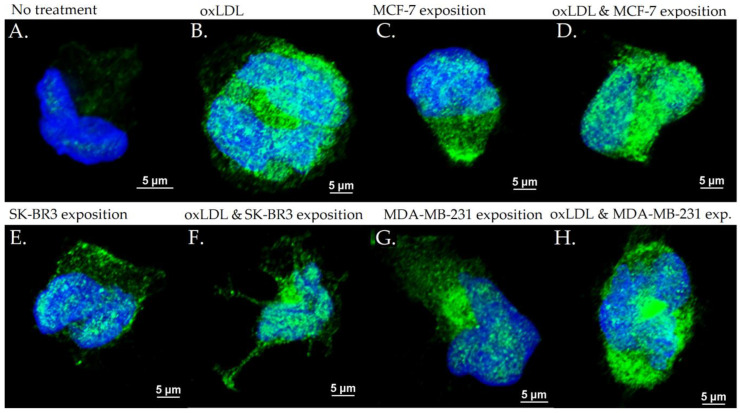
LOX-1 expression in THP-1 cells with (**A**) no treatment, (**B**) oxLDL exposure for 48 h, (**C**) 30% MCF-7 medium exposure for 48 h, (**D**) oxLDL plus 30% MCF-7 medium exposure for 48 h, (**E**) 30% SK-BR3 medium exposure for 48 h, (**F**) oxLDL plus 30% SK-BR3 medium exposure for 48 h, (**G**) 30% MDA-MB-231 medium exposure for 48 h, and (**H**) oxLDL plus 30% MDA-MB-231 medium exposure for 48 h (*n* = 10).

The exposition of the breast cancer cell medium on THP-1 for 48 h results in a significant increase of scavenger receptor expression. Indeed, we observed an LOX-1 MFI of 8.23 for non-treated cells (NT). The oxLDL condition shows an MFI of 31.74 (*t*-test, *p* < 0.001), MCF-7 exposure shows an MFI of 23.66 (*t*-test, *p* < 0.001), SK-BR3 exposure shows an MFI of 27.71 (*t*-test, *p* < 0.001), and MDA-MB-231 exposure shows an MFI of 28.13 (*t*-test, *p* < 0.001). When adding oxLDL to MCF-7 medium exposure, MFI was 27.46 (*t*-test, *p* < 0.001), while oxLDL in the SK-BR3 condition shows an MFI of 30.94 (*t*-test, *p* < 0.001) and oxLDL in the MDA-MB-231 condition shows an MFI of 32.96 (*t*-test, *p* < 0.001). No significant differences were highlighted between MCF-7, SK-BR3, and MDA-MB-231 and the condition with the addition of oxLDL (*t*-test, respectively, *p* = 0.128, *p* = 0.462, and *p* = 0.292 (Figure 7A and Figure 8).

**Figure 9:**
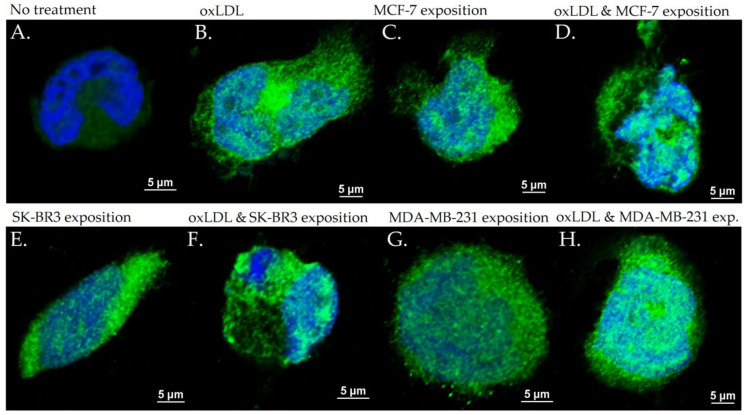
CD36 expression in THP-1 cells with (**A**) no treatment, (**B**) oxLDL exposure for 48 h, (**C**) 30% MCF-7 medium exposure for 48 h, (**D**) oxLDL plus 30% MCF-7 medium exposure for 48 h, (**E**) 30% SK-BR3 medium exposure for 48 h, (**F**) oxLDL plus 30% SK-BR3 medium exposure for 48 h, (**G**) 30% MDA-MB-231 medium exposure for 48 h, and (**H**) oxLDL plus 30% MDA-MB-231 medium exposure for 48 h (*n* = 10).

Regarding the CD36 expression, NT cells show an MFI of 8.67 whereas oxLDL exposition shows an MFI of 30.74 (*t*-test, *p* < 0.001). MCF-7 exposure shows an MFI of 28.04 (*t*-test, *p* < 0.001), SK-BR3 exposure shows an MFI of 30.84 (*t*-test, *p* < 0.001), and MDA-MB-231 shows an MFI of 27.70 (*t*-test, *p* < 0.001). When adding oxLDL to the MCF-7 medium exposure, we observed an MFI of 30.59 (*t*-test, *p* < 0.001). oxLDL added to the SK-BR3 medium shows an MFI of 30.12 (*t*-test, *p* < 0.001) whereas oxLDL to the MDA-MB-231 medium shows an MFI of 33.67 (*t*-test, *p* < 0.001). No significant differences were highlighted between MCF-7, SK-BR3, and MDA-MB-231 and the condition with the addition of oxLDL (*t*-test, respectively, *p* = 0.539, *p* = 0.882, and *p* = 0.134) (Figure 7B and Figure 9).

Regarding the CD162 expression which forms the endothelial adhesive markers, NT cells show an MFI of 0.33 whereas oxLDL exposition shows an MFI of 5.16 (*t*-test, *p* < 0.001). MCF-7 exposure shows an MFI of 1.557 (*t*-test, *p* = 0.002), SK-BR3 exposure shows an MFI of 3.87 (*t*-test, *p* = 0.008), and MDA-MB-231 shows an MFI of 5.69 (*t*-test, *p* < 0.001). When adding oxLDL to the MCF-7 medium exposure, we observed an MFI of 7.42 (*t*-test, *p* < 0.001).

**Figure 10:**
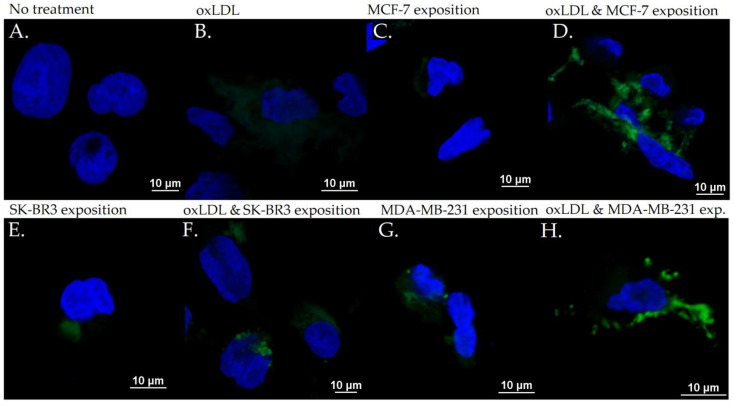
CD162 expression in THP-1 cells with (**A**) no treatment, (**B**) oxLDL exposure for 48 h, (**C**) 30% MCF-7 medium exposure for 48 h, (**D**) oxLDL plus 30% MCF-7 medium exposure for 48 h, (**E**) 30% SK-BR3 medium exposure for 48 h, (**F**) oxLDL plus 30% SK-BR3 medium exposure for 48 h, (**G**) 30% MDA-MB-231 medium exposure for 48 h, and (**H**) oxLDL plus 30% MDA-MB-231 medium exposure for 48 h (*n* = 10).

oxLDL added to the SK-BR3 medium shows an MFI of 6.42 (*t*-test, *p* = 0.001) whereas oxLDL added to the MDA-MB-231 medium shows an MFI of 8.88 (*t*-test, *p* < 0.001). There are no significant differences between the MDA-MB-231 and MDA-MB-231 plus oxLDL conditions (*t*-test, *p* = 0.083). However, adding oxLDL to MCF-7 and SK-BR3 reached statistical significance (*t*-test, respectively, *p* < 0.001, and *p* = 0.16) (Figure 7C and Figure 10).

## 4. Discussion

These results represent a significant advancement in our understanding of the impact of breast cancer on the initiation of atherosclerosis. We hypothesize that the effect of breast cancer on endothelial cells is mediated by the release of pro-inflammatory cytokines and reactive oxygen species (ROS) into the medium. However, despite achieving statistical significance, the observed increase in the fluorescence intensity of CD162 receptors after MCF-7 incubation is less pronounced. This lack of effect may be attributed to the expression of estrogen and progesterone in MCF-7 cells. Supporting this hypothesis, Rathod et al. reported a suppressed inflammatory leukocyte recruitment in women during the inflammatory state, resulting in reduced CD162 expression as observed through immunofluorescence [30].

Due to the overexpression of adhesive markers in endothelial cells and their corresponding ligands in circulating monocytes, the adhesion between these cells, which represents the initial stage of atherosclerosis, could be enhanced. Once monocytes enter into the sub-endothelium, they could be more easily transformed into foam cells with the overexpression of LOX-1 and CD36 at their surface [31]. Indeed, in healthy macrophages, the impact of LOX-1 on oxLDL uptake is approximately 5–10%, while, when LOX-1 is overexpressed, its ability to internalize oxLDL increases significantly, reaching up to 40% [32].

The phenotype of macrophage polarization should also be taken into consideration. Classically activated macrophages (M1) can be summarized as having a pro-inflammatory effect, which is pro-atherogenic, in comparison to the alternatively activated macrophages (M2) having an anti-inflammatory effect and, thus, an atheroprotective effect [33].

The proportion of the M2 phenotype is relatively higher in stable plaque than the M1 phenotype, but when the plaque becomes vulnerable, the proportion of M1 phenotype increases [34,35], and the M2 phenotype is less able to accumulate lipids due to a decreased expression of Liver-X-receptor α (LXRα), ATP-binding cassette transporter 1 (ABCA1), and Apolipoprotein E (ApoE) [36].

In the context of cancer, M1 macrophages recognize and destruct cancer cells, and their presence usually indicates a good prognosis [37]. Inversely, the tumor microenvironment is able to polarize M1 macrophages into M2 macrophages, in order to promote tumor growth [38]. Regarding these findings, it could be interesting to observe macrophage phenotypes on our THP-1 cells after breast cancer cell medium exposition, and characterize their pro- or anti-inflammatory phenotype, as observed by Cate et al. [39].

Finally, these results should be developed on a three-dimensional (3D) model to mimic an environment closer to the in vivo situation. These 3D models are more and more studied to understand the interplay between cancer and other pathologies. Some authors have decided to use a 3D spheroid model, as with Nguyen et al., who studied the anti-inflammatory effect of fluocinolone acetonide and dexamethasone on foam cell formation under a spheroid model [40]. Other authors studied the in vitro 3D cell-laden collagen hydrogel constructs which are composed of growth factors, hydrogels, and cells, providing a 3D in vitro environment beneficial for studying atherosclerosis [41]. Chen et al. studied the influence of smooth muscle cells (SMCs) on endothelial cell and monocyte adhesion, and concluded that the endothelial cells and SMC co-culture induced an increased leukocyte adhesion [42]. Recently, the improvements of tissue engineering enable the fabrication of tissue-engineered blood vessels (TEBVs) for studying atherogenesis [41]. Ragaseema et al. developed two-layered TEBVs by seeding endothelial cells and SMC on a biodegradable copolymer conduit in a perfusion system to obtain similar mechanical properties to native arteries [43], while Robert et al. fabricated a TEBV by seeding vascular cells and adding LDL and TNFα under high or low shear stress, demonstrating a preferential monocyte transmigration for the diseased model [44]. Finally, vessel-on-a-chip models have been developed to study atherosclerosis using a microfluidic platform including an engineered architecture recapitulating the microphysiological environment and architectures of functional human organs, connected to a pump to control flow rates and shear stress [45,46]. Poussin et al. used this model to study monocyte adhesion to human coronary artery endothelial cells, showing a significant increase in ICAM-1 expression after TFN-alpha treatment [47].

The limitation of this study lies in the method of LDL oxidation, which is induced by copper oxidation and may not accurately reflect the physiological conditions. Further experiments should be conducted using oxidation methods that better mimic physiological processes, such as myeloperoxidation. Additionally, it is important to note that our cell models were not exposed to shear stress, which is known to play a significant role in atherosclerosis, particularly at arterial bifurcations [48].

In conclusion, our study successfully demonstrated the enhanced oxidation of LDL upon contact with breast cancer cells, which was accompanied by an increased expression of receptors involved in the initiation of atherosclerosis. These findings provide new insights into the clinically observed interplay between atherosclerosis and cancer. Moving forward, additional research efforts should be undertaken to validate these hypotheses. This includes conducting in vitro experiments using microfluidic platforms to assess the effect of shear stress, as well as in vivo studies to investigate these associations in real-life human conditions. Despite the progress made in understanding the link between these two entities, there is still a significant knowledge gap that requires further investigations.

## Figures and Tables

**Table 1:** Summary of the different types of cells used in the following steps.

Cell Line	Cell Origin	Cell Markers	Culture Medium
SK-BR3	human breast adenocarcinoma	HER2-Neu + Oestrogen − Progesterone −	DMEM + 10% FBS + 2% L-Glutamin + 1% Penicillin/Streptomycin + 1% non-essential amino acids
MCF-7	human breast ductal carcinoma	HER2/Neu − Oestrogen + Progesterone +	See SK-BR3
MDA-MB-231	human breast adenocarcinoma	HER2-Neu − Oestrogen − Progesterone −	See SK-BR3
HUVEC	human umbilical vein endothelial cell		Endothelial Cell Growth Base Media + Endothelial Cell Growth Supplement + 1% Penicillin/Streptomycin
THP-1	human leukaemia monocytic cell		RPMI + 10% FBS + 5% L-Glutamin + 1% Penicillin/Streptomycin

## Data Availability

Not applicable.

## References

[1] Cardiovascular Diseases. https://www.who.int/health-topics/cardiovascular-diseases.

[2] Cancer. http://www.who.int/news-room/fact-sheets/detail/cancer.

[3] Dafni U., Tsourti Z., Alatsathianos I. (2019). Breast Cancer Statistics in the European Union: Incidence and Survival across European Countries. Breast Care.

[4] Ferlay J., Colombet M., Soerjomataram I., Dyba T., Randi G., Bettio M., Gavin A., Visser O., Bray F. (2018). Cancer incidence and mortality patterns in Europe: Estimates for 40 countries and 25 major cancers in 2018. Eur. J. Cancer.

[5] Carioli G., Malvezzi M., Rodriguez T., Bertuccio P., Negri E., La Vecchia C. (2017). Trends and predictions to 2020 in breast cancer mortality in Europe. Breast.

[6] Roubín S.R., Cordero A. (2019). The Two-way Relationship Between Cancer and Atherosclerosis. Rev. Española Cardiol. (Engl. Ed.).

[7] Florido R., Daya N.R., Ndumele C.E., Koton S., Russell S.D., Prizment A., Blumenthal R.S., Matsushita K., Mok Y., Felix A.S. (2022). Cardiovascular Disease Risk Among Cancer Survivors. J. Am. Coll. Cardiol..

[8] Kattoor A.J., Pothineni N.V.K., Palagiri D., Mehta J.L. (2017). Oxidative Stress in Atherosclerosis. Curr. Atheroscler. Rep..

[9] Rafieian-Kopaei M., Setorki M., Doudi M., Baradaran A., Nasri H. (2014). Atherosclerosis: Process, Indicators, Risk Factors and New Hopes. Int. J. Prev. Med..

[10] Tapia-Vieyra J.V., Delgado-Coello B., Mas-Oliva J. (2017). Atherosclerosis and Cancer; A Resemblance with Far-reaching Implications. Arch. Med Res..

[11] Murdocca M., De Masi C., Pucci S., Mango R., Novelli G., Di Natale C., Sangiuolo F. (2021). LOX-1 and cancer: An indissoluble liaison. Cancer Gene Ther..

[12] Galkina E., Ley K. (2007). Vascular Adhesion Molecules in Atherosclerosis. Arterioscler. Thromb. Vasc. Biol..

[13] Woollard K.J., Geissmann F. (2010). Monocytes in atherosclerosis: Subsets and functions. Nat. Rev. Cardiol..

[14] Katayama Y., Hidalgo A., Furie B.C., Vestweber D., Furie B., Frenette P.S. (2003). PSGL-1 participates in E-selectin–mediated progenitor homing to bone marrow: Evidence for cooperation between E-selectin ligands and α4 integrin. Blood.

[15] Faxon D.P., Fuster V., Libby P., Beckman J.A., Hiatt W.R., Thompson R.W., Topper J.N., Annex B.H., Rundback J.H., Fabunmi R.P. (2004). Atherosclerotic Vascular Disease Conference. Circulation.

[16] Kzhyshkowska J., Neyen C., Gordon S. (2012). Role of macrophage scavenger receptors in atherosclerosis. Immunobiology.

[17] Wingo P.A., Ries L.A.G., Rosenberg H.M., Miller D.S., Edwards B.K. (1998). Cancer incidence and mortality, 1973–1995. Cancer.

[18] Rotheneder M., Kostner G.M. (1989). Effects of low- and high-density lipoproteins on the proliferation of human breast cancer cellsIn vitro: Differences between hormone-dependent and hormone-independent cell lines. Int. J. Cancer.

[19] Dos Santos C.R., Fonseca I., Dias S., De Almeida J.C.M. (2014). Plasma level of LDL-cholesterol at diagnosis is a predictor factor of breast tumor progression. BMC Cancer.

[20] Khaidakov M., Mehta J.L. (2012). Oxidized LDL Triggers Pro-Oncogenic Signaling in Human Breast Mammary Epithelial Cells Partly via Stimulation of MiR-21. PLoS ONE.

[21] Pucci S., Polidoro C., Greggi C., Amati F., Morini E., Murdocca M., Biancolella M., Orlandi A., Sangiuolo F., Novelli G. (2019). Pro-oncogenic action of LOX-1 and its splice variant LOX-1Δ4 in breast cancer phenotypes. Cell Death Dis..

[22] Klooster C.C.V., Ridker P.M., Hjortnaes J., van der Graaf Y., Asselbergs F.W., Westerink J., Aerts J.G.J.V., Visseren F.L.J. (2019). The relation between systemic inflammation and incident cancer in patients with stable cardiovascular disease: A cohort study. Eur. Heart J..

[23] Raza U., Asif M.R., Bin Rehman A., Sheikh A. (2018). Hyperlipidemia and hyper glycaemia in Breast Cancer Patients is related to disease stage. Pak. J. Med. Sci..

[24] Pope A., Thomson L., Cantu S., Setia G., Torosyan N., Merz N.B., Atkins K., Anderson E.M., Cheng S., Tamarappoo B. (2022). Detection of subclinical atherosclerosis from PET-CT in patients with breast cancer. J. Cardiovasc. Comput. Tomogr..

[25] Cauley J.A., Zmuda J.M., Lui L.-Y., Hillier T.A., Ness R.B., Stone K.L., Cummings S.R., Bauer D.C. (2003). Lipid-lowering drug use and breast cancer in older women: A prospective study. J. Women’s Health.

[26] Bonovas S., Filioussi K., Tsavaris N., Sitaras N.M. (2005). Use of statins and breast cancer: A meta-analysis of seven randomized clinical trials and nine observational studies. J. Clin. Oncol..

[27] Scalia A., Kindt N., Trelcat A., Nachtergael A., Duez P., Journé F., Carlier S. (2022). Development of a Method for Producing oxLDL: Characterization of Their Effects on HPV-Positive Head and Neck Cancer Cells. Int. J. Mol. Sci..

[28] Holliday D.L., Speirs V. (2011). Choosing the right cell line for breast cancer research. Breast Cancer Res..

[29] Genin M., Clement F., Fattaccioli A., Raes M., Michiels C. (2015). M1 and M2 macrophages derived from THP-1 cells differentially modulate the response of cancer cells to etoposide. BMC Cancer.

[30] Rathod K.S., Siddiqui U., Hartley A., Khambata R., Ahluwalia A. (2016). Investigation of the influence of sex on cantharidin-induced inflammation in healthy volunteers. FASEB J..

[31] Li D., Mehta J.L. (2009). Intracellular signaling of LOX-1 in endothelial cell apoptosis. Circ. Res..

[32] Schaeffer D.F., Riazy M., Parhar K.S., Chen J.H., Duronio V., Sawamura T., Steinbrecher U.P. (2009). LOX-1 augments oxLDL uptake by lysoPC-stimulated murine macrophages but is not required for oxLDL clearance from plasma. J. Lipid Res..

[33] Yao Y., Xu X.-H., Jin L. (2019). Macrophage Polarization in Physiological and Pathological Pregnancy. Front. Immunol..

[34] Stöger J.L., Gijbels M.J., van der Velden S., Manca M., van der Loos C.M., Biessen E.A., Daemen M.J., Lutgens E., de Winther M.P. (2012). Distribution of macrophage polarization markers in human atherosclerosis. Atherosclerosis.

[35] Bisgaard L.S., Mogensen C.K., Rosendahl A., Cucak H., Nielsen L.B., Rasmussen S.E., Pedersen T.X. (2016). Bone marrow-derived and peritoneal macrophages have different inflammatory response to oxLDL and M1/M2 marker expression—Implications for atherosclerosis research. Sci. Rep..

[36] Gleissner C.A., Shaked I., Little K.M., Ley K. (2010). CXC Chemokine Ligand 4 Induces a Unique Transcriptome in Monocyte-Derived Macrophages. J. Immunol..

[37] Weagel E., Smith C., Liu P.G., Robison R., O’neill K. (2015). Macrophage Polarization and Its Role in Cancer. J. Clin. Cell. Immunol..

[38] Guiducci C., Vicari A.P., Sangaletti S., Trinchieri G., Colombo M.P. (2005). Redirecting in vivo elicited tumor infiltrating macrophages and dendritic cells towards tumor rejection. Cancer Res..

[39] Pe K.C.S., Saetung R., Yodsurang V., Chaotham C., Suppipat K., Chanvorachote P., Tawinwung S. (2022). Triple-negative breast cancer influences a mixed M1/M2 macrophage phenotype associated with tumor aggressiveness. PLoS ONE.

[40] Nguyen L.T.H., Muktabar A., Tang J., Wong Y.S., Thaxton C.S., Venkatraman S.S., Ng K.W. (2018). The Potential of Fluocinolone Acetonide to Mitigate Inflammation and Lipid Accumulation in 2D and 3D Foam Cell Cultures. BioMed Res. Int..

[41] Chen J., Zhang X., Millican R., Lynd T., Gangasani M., Malhotra S., Sherwood J., Hwang P.T., Cho Y., Brott B.C. (2022). Recent Progress in in vitro Models for Atherosclerosis Studies. Front. Cardiovasc. Med..

[42] Chen C.-N., Chang S.-F., Lee P.-L., Chang K., Chen L.-J., Usami S., Chien S., Chiu J.-J. (2006). Neutrophils, lymphocytes, and monocytes exhibit diverse behaviors in transendothelial and subendothelial migrations under coculture with smooth muscle cells in disturbed flow. Blood.

[43] Ragaseema V.M., Columbus S., Ramesh R., Krishnan L. (2013). Potential of Tissue Engineered Blood Vessel as Model to Study Effect of Flow and Wall Thickness on Cellular Communication. Curr. Tissue Eng..

[44] Robert J., Weber B., Frese L., Emmert M.Y., Schmidt D., von Eckardstein A., Rohrer L., Hoerstrup S.P. (2013). A Three-Dimensional Engineered Artery Model for In Vitro Atherosclerosis Research. PLoS ONE.

[45] Zheng F., Fu F., Cheng Y., Wang C., Zhao Y., Gu Z. (2016). Organ-on-a-Chip Systems: Microengineering to Biomimic Living Systems. Small.

[46] Cochrane A., Albers H.J., Passier R., Mummery C.L., van den Berg A., Orlova V.V., van der Meer A.D. (2019). Advanced in vitro models of vascular biology: Human induced pluripotent stem cells and organ-on-chip technology. Adv. Drug Deliv. Rev..

[47] Poussin C., Kramer B., Lanz H.L., Heuvel A.V.D., Laurent A., Olivier T., Vermeer M., Peric D., Baumer K., Dulize R. (2020). 3D human microvessel-on-a-chip model for studying monocyte-to-endothelium adhesion under flow—Application in systems toxicology. ALTEX-Altern. Anim. Exp..

[48] Heo K.-S., Fujiwara K., Abe J.-I. (2014). Shear stress and atherosclerosis. Mol. Cells.

